# The environmental-data automated track annotation (*Env*-*DATA*) system: linking animal tracks with environmental data

**DOI:** 10.1186/2051-3933-1-3

**Published:** 2013-07-03

**Authors:** Somayeh Dodge, Gil Bohrer, Rolf Weinzierl, Sarah C Davidson, Roland Kays, David Douglas, Sebastian Cruz, Jiawei Han, David Brandes, Martin Wikelski

**Affiliations:** Department of Civil, Environmental & Geodetic Engineering, The Ohio State University, 475 Hitchcock Hall, 2070 Neil Avenue, Columbus, USA; Max Planck Institute for Ornithology, Radolfzell, Germany; NC Museum of Natural Sciences, NC State University, Raleigh, USA; U.S. Geological Survey, Alaska Science Center, Juneau, USA; University of Illinois at Urbana-Champaign, Urbana, USA; Lafayette College, Easton, USA; Department of Biology, University of Konstanz, Konstanz, Germany

**Keywords:** Animal movement, Migration, Movebank, Movement ecology, Remote sensing, Track annotation, Weather

## Abstract

**Background:**

The movement of animals is strongly influenced by external factors in their surrounding environment such as weather, habitat types, and human land use. With advances in positioning and sensor technologies, it is now possible to capture animal locations at high spatial and temporal granularities. Likewise, scientists have an increasing access to large volumes of environmental data. Environmental data are heterogeneous in source and format, and are usually obtained at different spatiotemporal scales than movement data. Indeed, there remain scientific and technical challenges in developing linkages between the growing collections of animal movement data and the large repositories of heterogeneous remote sensing observations, as well as in the developments of new statistical and computational methods for the analysis of movement in its environmental context. These challenges include retrieval, indexing, efficient storage, data integration, and analytical techniques.

**Results:**

This paper contributes to movement ecology research by presenting a new publicly available system, Environmental-Data Automated Track Annotation (*Env*-*DATA*), that automates annotation of movement trajectories with ambient atmospheric observations and underlying landscape information. *Env*-*DATA* provides a free and easy-to-use platform that eliminates technical difficulties of the annotation processes and relieves end users of a ton of tedious and time-consuming tasks associated with annotation, including data acquisition, data transformation and integration, resampling, and interpolation. The system is illustrated with a case study of Galapagos Albatross (*Phoebastria irrorata*) tracks and their relationship to wind, ocean productivity and chlorophyll concentration. Our case study illustrates why adult albatrosses make long-range trips to preferred, productive areas and how wind assistance facilitates their return flights while their outbound flights are hampered by head winds.

**Conclusions:**

The new *Env*-*DATA* system enhances Movebank, an open portal of animal tracking data, by automating access to environmental variables from global remote sensing, weather, and ecosystem products from open web resources. The system provides several interpolation methods from the native grid resolution and structure to a global regular grid linked with the movement tracks in space and time. The aim is to facilitate new understanding and predictive capabilities of spatiotemporal patterns of animal movement in response to dynamic and changing environments from local to global scales.

**Electronic supplementary material:**

The online version of this article (doi:10.1186/2051-3933-1-3) contains supplementary material, which is available to authorized users.

## Background

The movement of an organism is influenced not only by its internal state and biological factors driving its movement, but also external factors—the environment and underlying context [1]. Environmental conditions may trigger certain movement patterns or invoke a particular behavioral response, and thus determine local movements or long-distance migrations [2–4]. Animals can optimize their energy expenditure during movement by selecting for locations and times when the conditions are supportive for movement. For example, raptors in their southward fall migration select a preferential mode of uplift that best fits their flight capacity [5–7]. Spatial and temporal variability in environmental conditions may affect all types of movement and any scale, from local to global. Some of the most challenging movements to study include large-scale movement, such as migrations and movements that cross broad geographic areas and traverse diverse environments and landscapes. The added challenge in these studies is that environmental conditions cannot be measured locally as part of the study, because they are needed over a very extensive area. Long-distance migrants include some of our most endangered species, and thus it is critical to address questions at the core of movement ecology, such as “when do animals start migrating?”, “which strategies should animals adopt while migrating?” and “do movement rules change in a changing environment, and if so, how?” [8].

Today, with the rapid improvement and miniaturization of tracking technology, movement ecology has entered a new data-rich era, with tremendous growth in animal tracking data at previously unseen spatial and temporal resolution. Complementing this are large arrays of online remote sensing datasets describing the earth system and informing models that forecast the future environment. Combining these datasets is an active area of research, addressing a variety of questions to gain a better understanding of the interaction between animal movement and the environment. Manual annotation of animal tracking data (i.e. adding information to locations by an expert) and simulations of the environment along movement tracks have been successfully used to discover meaningful interactions between movement and external variables [2, 5, 7, 9–25]. Improving access to these environmental data will increase our understanding of their broad effects on our planet, motiving the development of RNCEP, a data organization and visualization package for R for working with data from National Centers for Environmental Prediction (NCEP) / National Center for Atmospheric Research (NCAR) Reanalysis data [26].

Manual annotation is not practical for large global environmental datasets owing to several technical and logistical challenges. The remote sensing datasets needed to study how environmental conditions influence animal movements are provided using complex tiling system in space and time that need to be aggregated to cover the entire movement track. Environmental and animal movement data are usually collected in different spatial and temporal scales and it is therefore necessary to choose appropriate scales for the annotation process. Likewise, an appropriate interpolation technique must be applied in order to integrate data at different resolutions. Moreover, environmental data are diverse in source, format, and projection system. It is essential to apply appropriate data transformation techniques in order to integrate such heterogeneous datasets. Accordingly, effective storage, indexing, and retrieval strategies must be applied to handle large volume of environmental datasets. These challenges limit many potential non-technical users from accessing these data and applying annotation in a manual mode. Nonetheless, compiling combined movement-environment datasets would be highly beneficial for movement studies. Such vast datasets are well suited for sophisticated, context-aware data mining and pattern recognition techniques that allow researchers to discover patterns of movement in response to changes in the environment [27, 28]. Hence, an integrated system capable of managing and analyzing movement tracks of animals linked to large remote sensing, climatic, and land use datasets will greatly facilitate the next generation of research into movement ecology.

This paper contributes to movement ecology research by describing a new open system, Environmental-Data Automated Track Annotation (*Env*-*DATA*), that automates annotation of movement trajectories with ambient atmospheric observations and underlying landscape information. The aim of the system is to provide efficient movement track annotation and knowledge discovery methods to allow scientists to examine relationships between observed animal movements and a breadth of information about environmental conditions. The *Env*-*DATA* system utilizes large computational servers to co-register the animal tracks with environmental data without requiring the user be an expert in the processing of such data. The system facilitates the investigation of biological research questions about movement behavior of animals, including threatened and endangered species that are of concern due to the impact of climate and environmental changes. *Env*-*DATA* will facilitate discovery of unique information about niche selection and habitat, movement patterns and landscape connectivity of moving animals, and how these may be affected by variability and long-term changes in climate and landscape. Such knowledge is crucial for planning and management of protected areas and for forecasting population status and habitat needs in future conditions of climate and land use changes.

## Methods

### The movebank *Env*-*DATA* system

**Figure 1:**
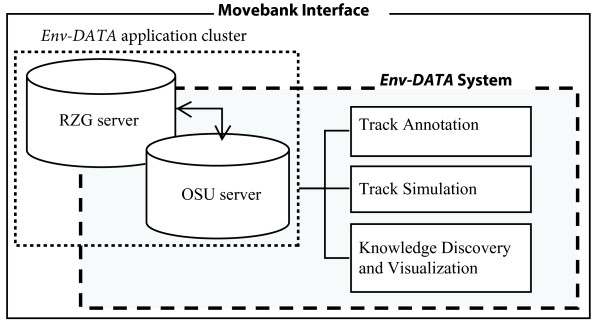
**Movebank** ***Env***-***DATA*** **System.** RZG: Computing Center Garching, Germany; OSU: The Ohio State University Supercomputer Center. The gray box highlights the *Env*-*DATA* system components within Movebank.

This paper primarily focuses on the architecture and technical characteristics of the *Env*-*DATA* Track Annotation service. The *Env*-*DATA* system expands the capabilities of Movebank, an animal movement data portal (http://www.movebank.org). Movebank is a free, online database of animal tracking data, which provides biologists and animal movement researchers with a secure online archive to store, manage, process, and share animal movement data [29, 30]. Figure 1 illustrates the main components of the Movebank *Env*-*DATA* system. The *Env*-*DATA* system extensions within Movebank include three main services: (1) the Track Annotation Service, (2) the Track Simulation Service, and (3) the Knowledge Discovery and Visualization Service.

The Track Simulation and the Knowledge Discovery and Visualization services are developed as external libraries using the Java^(TM)^ Platform and the R programming language. These libraries can read tracking data directly from Movebank (provided the authenticated user has the necessary access rights), and read-write annotated data that were prepared by the Track Annotation Service. They were developed to serve as a convenient access layer to *Env*-*DATA* and can be used to transfer data to any other programming or data analysis environment. They can, for example, directly communicate and exchange data with common analysis, visualization and simulation tools like R, MATLAB and Google Earth, and niche analysis software such as Maxent [31]. Examples for such applications using R and Google Earth are described in the case study section below. In addition, several open movement analysis packages providing tools for knowledge discovery, data mining, modeling, and visualization have been developed by others, including the Move R-package [32]—a movement track analysis and visualization package— and MoveMine [33]—a track segmentation and classification package.

To ensure its relevance and effectiveness, Movebank services and the *Env*-*DATA* system were designed and tested in collaboration with several wildlife research partners from the U.S. Fish and Wildlife Service (FWS), the U.S. National Park Service (NPS), and the U.S. Geological Survey (USGS), who contributed to the design of *Env*-*DATA* to ensure its applicability and relevance to contemporary conservation and wildlife management [34–36].

### System infrastructure

**Figure 2:**
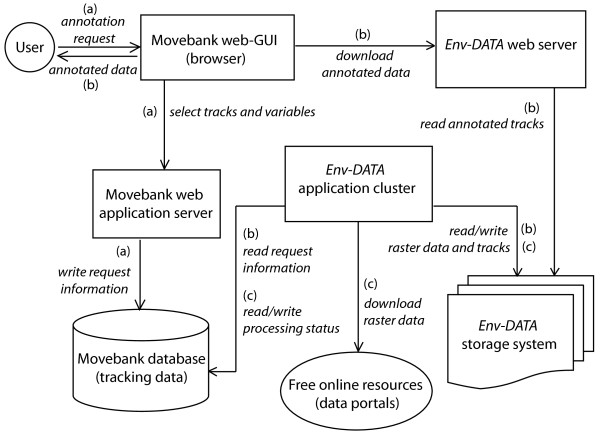
***Env***-***DATA*** **Track Annotation Service Components.** The figure illustrates the workflow of an annotation request through the different servers and components of the system. Steps shown indicate the following: (a) selection and submission of a data annotation request by the User, (b) read annotation request information, process Track Annotation in the *Env*-*DATA* application cluster, storage of annotation results in the *Env*-*DATA* storage system, and delivery of results to User through the *Env*-*DATA* web server, and (c) environmental data acquisition and storage in the *Env*-*DATA* storage system through the *Env*-*DATA* application cluster. RZG: Computing Center Garching, Germany; OSU: The Ohio State University Supercomputer Center.

The system runs on a Linux application cluster and manages data flow using a MySQL database. Two technically identical installations of the *Env*-*DATA* application cluster are located at the Computing Center Garching (RZG) of the Max Planck Society in Garching, Germany, and at the Ohio Supercomputer Center (OSC) of The Ohio State University in Columbus (OH, USA). They serve as storage and processing systems for the environmental data cached directly from their original data sources. Each cluster (i.e. at RZG and OSC) serves different sets of environmental variables depending on their proximity to the original data source. The Movebank application server, which serves the animal tracking data, is also located at the RZG (Figures 1 and 2).

### Environmental data

Our goal is to provide access to the most relevant global gridded datasets of environmental data. Datasets that are available in this first release of *Env*-*DATA* include NASA’s MODIS vegetation, ocean, ice and fire data products; NCEP Global and North American Regional Reanalyses (NARR); the European Centre for Medium-Range Weather Forecasts (ECMWF) moderate resolution reanalysis; Oregon State University Ocean Net Primary Productivity (NPP); NOAA’s Ocean Surface Current Analyses (OSCAR) ocean currents and sea surface temperatures; NASA’s Tropical Rainfall Measuring Mission (TRMM) precipitation; normalized difference vegetation index from the AVHRR sensor; ESA’s GlobeCover land use and land cover; NASA’s ASTERGDEM 30 m topography; and the Columbia University Human Geography dataset. The raw data are obtained in different formats, such as NetCDF, GRIB, HDF, GeoTIFF, and ASCII. See Table 1 for more details about the datasets. *Env*-*DATA* also offers derived variables that are particularly suited to aerial movement studies, such as tail-wind support and uplift availability (Table 1).

**Table 1:** **Available environmental datasets for the trajectory annotation service**

Datasets	Data Description	Data Source	Projection system/Grid	Temporal Coverage	Geographic coverage (Latitude/Longitude)	Temporal resolution	Spatial resolution	Data Format
Tropical Rainfall Measuring Mission (TRMM) [37]	Tropical precipitation	NASA http://trmm.gsfc.nasa.gov/	Regular lat/lon grid	1998– present	50°N–50°S 180°E–180°W	3-hour	0.25°	Unformatted binary
AVHRR land NDVI [38]	Normalized difference vegetation index from the AVHRR (low resolution) sensor	USGS (USA only) http://phenology.cr.usgs.gov/get_data_1km.php NASA (global) http://glcf.umiacs.umd.edu/data/gimms/	Albert’s Equal Area grids	1989–present, 1982–present	CONUS, 90°N–90°S 180°E–180°W	1-week, 2-week	1 km (USA), 8 km (global)	Unformatted binary
NCEP Global Reanalysis 2 [39]	Global weather reanalysis	NOAA http://www.esrl.noaa.gov/psd/data/gridded/data.ncep.reanalysis2.html	Regular (non-Gaussian) grid	1948–present	90°N–90°S 180°E–180°W	6-hour	2.5° (208 km)	NetCDF
North American Regional Reanalysis (NARR) [40]	Regional (North America only) weather reanalysis	NOAA http://www.emc.ncep.noaa.gov/mmb/rreanl/	Lambert Conformal, Conic Grids	1979–present	90°N–1°N 0°–170W°	3-hour	32 km (at 40°N)	GRIB
ECMWF Reanalysis [41]	Global weather reanalysis	ECMWF http://www.ecmwf.int/	Regular grid	1979–present	89.463°N–89.463°S 180°E–180°W	6-hour	0.7°	GRIB
MODIS Land	Earth-surface, reflectivity and vegetation variables	NASA https://lpdaac.usgs.gov/	Geographic/ Sinusoidal grid	2002–2012	90°N–90°S 180°E–180°W	Daily, 8-day, 16-day, monthly	5.6 km (0.05°)	HDF- EOS
MODIS Ocean	Ocean surface, color, and productivity variables	NASA http://oceancolor.gsfc.nasa.gov/	Cylindrical Equidistant				4 km, 9 km	HDF- EOS
MODIS Snow	Snow and ice variables	NASA http://modis-snow-ice.gsfc.nasa.gov/	Cylindrical Equidistant				1 Km, 4 Km	HDF- EOS
Ocean productivity [42]	Ocean net primary productivity (NPP) reanalysis	http://www.science.oregonstate.edu/ocean.productivity/	Equidistant Cylindrical projection, lat/lon grid	1997–2009	90°N–90°S 180°E–180°W	8-day, monthly	Grid sizes 1080x2160 (1/6 degree) 2160x4320 (1/12 degree)	HDF
ASTER GDEM	Very high-resolution topography	USGS http://asterweb.jpl.nasa.gov/gdem.asp	Regular grid, (WGS84 ellipsoid)		83°N–83°S 180°E–180°W		1 arc-second	GeoTIFF
SRTM [43]	High resolution topography	NASA http://www.cgiar-csi.org/data/srtm-90m-digital-elevation-database-v4-1	Regular grid, (WGS84 ellipsoid)		60°N–60°S 180°E–180°W		3 arc-second	HGT
GlobCover	Land cover and land-use type	ESA http://dup.esrin.esa.it/prjs/prjs68.php	Plate-Carrée projection (WGS84 ellipsoid)	2009	90°N–65°S 180°E–180°W		20 arc-seconds	HDF
Socioeconomic data (Population Density Grid)	Human geography	http://sedac.ciesin.columbia.edu/gpw/global.jsp	Regular grid (WGS84 ellipsoid)	1990–2010	85°N–58°S 180°E–180°W	5 years	30 arc-second (1km)	ASCII
Ocean Surface Current Reanalysis (OSCAR)	Ocean surface currents	NASA http://www.oscar.noaa.gov/	Regular grid	1993–present	60°N–60°S 180°E–180°W	5-day, monthly	1 degree, 1/3 degree	NetCDF
ETOPO1	Ice surface and bedrock	NASA http://www.ngdc.noaa.gov/mgg/global/global.html	Regular grid (WGS84 ellipsoid)	1940–2008	90°N–90°S 180°E–180°W		1 arc-minute	NetCDF
Distance to the Nearest Coast	Distance to the nearest coast	NASA http://oceancolor.gsfc.nasa.gov/DOCS/DistFromCoast/	Regular grid		90°N–90°S 180°E–180°W		0.04° 0.01°	Text file, GeoTiff
Derived wind variables for flight	Tail-wind support and cross wind [36]; Thermal and orographic uplift [5]	Calculated derived variables, based on ECMWF or NCEP data	Regular grid	1979–present	89.463°N–89.463°S 180°E–180°W	6-hour	0.7°	ASCII
Derived topographic variables	Slope and aspect [25]; Rugosity [44]	Calculated derived variables, based on ASTERGDEM	Regular grid		83°N–83°S 180°E–180°W		1 arc-second	ASCII

### Track annotation service

The term “path annotation”, borrowed from computer science, is used when additional data about important variables encountered through a particular path are added to the dataset describing an object’s trajectory. In the context of animal movement, path (track) annotation includes environmental variables co-located in time and space with the moving organism’s coordinates [7]. The *Env*-*DATA* Track Annotation Service is the fundamental extension of the Movebank portal that attributes environmental data to each tracking location (in space and time) along a movement path. The service consists of several components as illustrated in Figure 2 and described below.

The *Env*-*DATA* application cluster resides on the RZG and OSC servers and is the main core of the system (Figures 1 and 2). Data flow required for the annotation service is handled through the Movebank web application server and *Env*-*DATA* web server using MySQL. The annotation service is triggered by a request from the User using the Movebank web-GUI (arrows (a) in Figure 2) and is processed at the *Env*-*DATA* application cluster. The annotation results are then stored in the *Env*-*DATA* storage system and an email notification, including an http download link, is sent to the user through the *Env*-*DATA* web server when data are available for download (arrows (b) in Figure 2). The *Env*-*DATA* web server is a dedicated machine that runs a Tomcat web application server and provides an http interface to the service running on the *Env*-*DATA* application cluster. The *Env*-*DATA* web server functionality is limited to accepting annotation requests, storing them in the database, and delivering the results. All data processing is performed on the *Env*-*DATA* application cluster, which is a family of Linux compute nodes.

The annotation service is conducted offline because of the large volumes of data involved. Latency of the service depends on the time required to download the necessary environmental data. The annotation workflow involves several steps, described below.

#### Annotation request

Users may request two types of annotation: (1) annotation of a gridded geographic area or (2) annotation of a set of trajectories. For trajectory annotations, the *output* spatial and temporal resolutions and extent are determined by the system according to the spatial and temporal constraints of the *input* trajectories.

**Figure 3:**
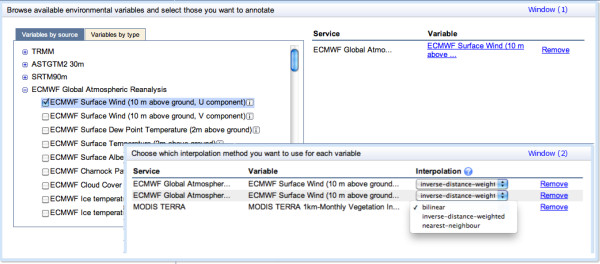
**An example for the graphical user interface (GUI) that serves the annotation system users.** The figure illustrates an annotation request for the data in the variable “surface wind (10m above ground, U component)” from the global weather reanalysis dataset ECMWF (see Table 1 for more details), and selection of interpolation methods for each requested variable.

A trajectory annotation request starts with the selection of one or more animal tracks from an existing user-created study in Movebank. After selecting the trajectories the user is asked to choose a subset of environmental variables (for example, window (1) in Figure 3). There are two methods (represented by separate tabs) for browsing and selecting variables (window (1) in Figure 3): (1) select variables by source, with environmental variables organized according to their original satellite missions or dataset portal (e.g. MODIS or ECMWF, see Table 1 for complete list); or (2) select variables by type, where the variables are organized according to their geophysical composition (e.g. weather, topography, earth surface and vegetation, ocean). Both methods lead the user through a hierarchical classification (e.g. weather → temperature → surface temperature → NCEP air temp at 10 m) to the point where specific variables can be selected and added to the annotation request. Summary information about each variable and links to source documentation are available from the interface. Next, the user chooses an interpolation method for each environmental variable (window (2) in Figure 3). The annotation service provides three interpolation methods: nearest neighbor, bilinear, and inverse weighted distance, as explained in “Data Interpolation” below.

For annotations of geographic areas, the user specifies the coordinates of four corners of a region of interest, a coordinate reference system (e.g. WGS84 lon/lat), required timestamps (e.g. as “yyyy-MM-dd HH:mm:ss”, comma separated), and a spatial resolution for the target grid (in number of pixels). Data will be interpolated if the requested resolution is higher than the native resolution of the dataset. The result is a bundle of mapped data that can be read for numerical analysis (for example by a niche model, such as Maxent), or used as GeoTiff images or as KML image overlays in Google Earth.

After selecting the desired information for either annotation method, the user provides an e-mail address to which a link to the completed data file will be mailed and submits the request.

#### Data acquisition

As the combined volume of globally available environmental data is on the order of petabytes, it is not feasible to locally mirror all of the source environmental data. Instead, the *Env*-*DATA* application cluster applies a caching strategy to retain the most frequently accessed data and download any other data upon request if it is not already locally stored at the *Env*-*DATA* storage system (arrows (c) in Figure 2). New data requests (provided as a list of locations, times and variables) are translated to lists of needed data sources, sorted according to data service, variable, timestamp and scene (i.e. a raster tile). Multiple data sources are listed when the location in the movement path requires interpolation between scenes and/or in time, or when derived variables (such as thermal uplift) require a combination of several input environmental variables. The data-sources list is compared with the stored metadata table and data that are not stored locally are requested from their provider using an ftp/http, or OPeNDAP interface. The system ranks each scene according to the frequency at which it has been accessed since download. The least accessed scenes are deleted when space is needed for new data.

#### Data retrieval, indexing, and transformation strategies

The environmental data are acquired in a variety of data formats (e.g. GeoTIFF, NetCDF, HDF, GRIB). Prior to the annotation, *Env*-*DATA* extracts the required variables from the original data structure using available Java libraries associated with the data formats. For each variable an *n*-dimensional grid is defined (*1* ≤ *n* ≤ *4*, possible dimensions are *x*, *y*, *z*, *t*) which spans the complete domain of definition of the variable. Each point in the grid is assigned a tile index and tile indices are mapped to the names of files that contain the data for a tile. When a set of points is submitted for annotation by the *Env*-*DATA* system, each point is transformed to the coordinate reference system of the annotation variable using proper projection techniques. A set of neighboring grid points is then determined and the names of the files containing data for these grid points are identified. For those files not already stored in the *Env*-*DATA* storage system, an asynchronous job is started to download them from the original source. When the system runs out of disc space for storing new files, a garbage collection job deletes files following a least recently used (LRU)-based algorithm. Track annotation and interpolation starts when all required files have been downloaded. Data files are read in blocks and blocks are cached in an in-memory LRU cache. The block structure is chosen to match the physical structure of the underlying file in order to optimize read performance. In order to optimize cache use, data points are sorted according to the files they require for annotation and the within-file block structure.

Separate processing steps (e.g. pre-processing, data download, garbage collection and track annotation) are performed in parallel on a pool of compute nodes at the *Env*-*DATA* application cluster, while resource access is coordinated by a bespoke locking system implemented on MySQL.

#### Data interpolation

**Figure 4:**
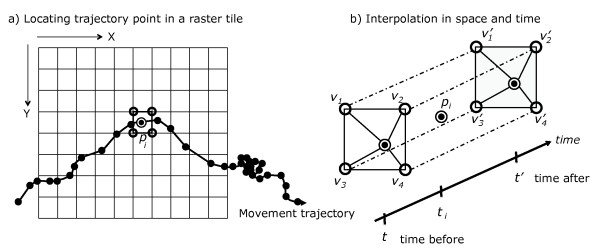
**Interpolation in space and time.** (**a**) The variable data for track-point *P*_*i*_ is first interpolated in space (using one of several interpolation methods) based on the data from the available points in the environmental dataset native grid around *P*_*i*_. (**b**) Similar spatial interpolations are conducted at the two nearest available points in time, the nearest before and nearest after the timestamp of the track-point *P*_*i*_. Then, the two interpolated spatial values are interpolated in time to the timestamp of *P*_*i*_.

Once all necessary data sources are locally available, the environmental data are interpolated along all trajectory points. Prior to the interpolation, the trajectories are first transformed to the native grid of the environmental variables (e.g. the Sinusoidal grid for MODIS or Lambert Conformal for NARR), if required. After the transformation, for each point along a trajectory, the interpolation first is applied in space, then in time. For each trajectory point *p*_*i*_ (*x*_*i*_, *y*_*i*_, *t*_*i*_,), four adjacent pixels (or more if necessary) are located in the global grid of environmental data in space (Figure 4a) at two temporal timestamps before and after *t*_*i*_ (Figure 4b). The values of the four neighbor pixels (i.e. *v*_*1*_…*v*_*4*_ and *v*_*1*_’…*v*_*4*_’) at two timestamps *t* and *t*’ are then extracted and used to compute an interpolated value for the trajectory point *p*_*i*_.

The system allows three types of interpolation in space: *nearest neighbor*, *bilinear*, and *inverse weighted distance*[45, 46], and two types in time: *nearest neighbor* and *inverse weighted distance*. The user can apply different methods of interpolation to space and to time. However, the available interpolation strategy differs according to the type of data. For instance, for categorical data, such as land-use type, only the nearest neighbor interpolation can be applied, whereas for continuous-numeric data types either a nearest neighbor, bilinear (in regular grids), or inverse weighted distance interpolation can be selected based on the resolution of data [45, 46]. Spatial distances used by the nearest neighbor and inverse weighted distance interpolations are calculated as great circle distances on the WGS84 ellipsoid.

#### Results delivery

The annotated trajectories are delivered to the user via http download in comma-separated values (csv) format, and gridded geographic areas as GeoTiff or KML images. The user receives an e-mail with a link when the download is ready. The annotated data are stored in the *Env*-*DATA* storage system at OSU and delivered to the user through the *Env*-*DATA* web server (cf. arrow (b) in Figure 2). Metadata for the output is uniform across different annotation requests and movement data types, which simplifies linking the output with further analysis tools. A user-group community in Movebank provides a place to exchange user-developed codes and statistical and analytical methods, suggest knowledge discovery and data mining methods, or comment on existing tools and research challenges.

## Case study: Galapagos albatross (*phoebastria irrorata*)

This case study illustrates an application of the *Env*-*DATA* System to examine environmental factors associated with the movements of nine Galapagos Albatrosses (*Phoebastria irrorata*), tracked from June to September 2008.

### Methods – movement data collection and annotation

The original tracking data were collected from birds breeding at two sites on Isla Española, Punta Cevallos (1.39° S, 89.62° W) and Punta Suarez (1.38° S, 89.75° W), as well as a small island close to the Ecuadorian mainland, Isla de la Plata (1.58° S, 81.15° W). The birds were tracked throughout the entire breeding season. GPS loggers were deployed on 28 adult albatrosses at the beginning of incubation period. The loggers weighed 22 g with a 9-month battery life and were designed and produced by e-obs GmbH (Munich, Germany). Units were programmed to record GPS locations every 90 minutes. Units were attached to taped bundles of dorsal feathers and secured with cable ties. Data from GPS units were downloaded remotely via an ultra-high frequency (UHF) radio link to a stationary base station that was installed in the vicinity of equipped bird nests at each separate colony. A base station consists of a UHF antenna, a receiver with flash memory and two 6 volt 12 amp-hour batteries. The base station begins to upload data when any unit (or equipped bird) comes within 1000 m of the station. This system has the advantage of allowing data to be retrieved automatically without physically recovering the tag, reducing handling-induced stress to the birds and labor required to collect data in the field. Every four weeks a researcher would go to sites to retrieve data from base stations, change batteries and perform general maintenance duties. On Isla Española, tags were deployed on 23 June 2008 at Punta Cevallos and 31 May 2008 at Punta Suarez, and data were retrieved from base stations on 23 June, 21 July, 12 August and 18 September 2008. On Isla de la Plata, tags were deployed on 7–24 June 2008, data were downloaded on 7 July, 4 August, 1 September, and 6 October of 2008, and loggers were recovered on 7 October.

For the purpose of this case study, we used tracking data from nine albatrosses that made extensive movements for almost the entire period from June to September 2008. The tracks were segmented to transit flights from/to the Galapagos Islands and the Peruvian coastal foraging segments. The transit flights are segmented according the flight speed obtained from GPS points (speed >5 m/s) between longitudes 90°W and 82.5°W. The Peruvian coastal foraging area is defined as areas between longitudes 82.5°W and 75°W. Using *Env*-*DATA*, the nine albatross tracks were annotated with wind speed (m/s) and wind direction (degrees from North) computed from u- and v-wind components obtained from the NCEP Reanalysis 2 dataset, and Ocean Net Primary Production (NPP) data from Oregon State University (Table 1). For the annotation 6-hour, 2.5° NCEP Reanalysis 2, and 8-day, 2160x4320 ocean NPP datasets are used (cf. Table 1).

## Results and discussion

**Figure 5:**
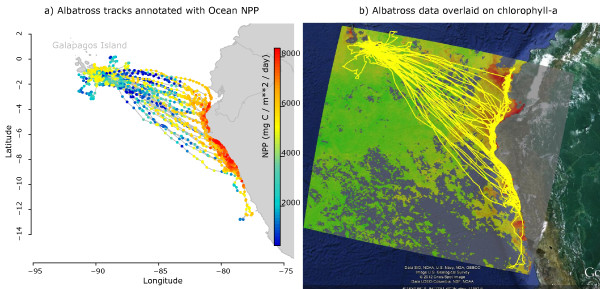
**Nine annotated albatross trajectories.** The tracks of nine adult albatrosses, overall containing 8286 GPS locations, during the breeding season in June to September 2008, (**a**) color coded with annotated values of 8-day ocean NPP (see Table 1 for more information on this variable), (**b**) the same tracks (yellow lines) plotted on the geographic area annotation using the monthly MODIS-ocean chlorophyll-a variable (Table 1) for the month of July 2008. We used the KML data format and combined the annotated area with a Google-Earth satellite image of the region using the program Matlab and its “Google Earth Toolbox”.

**Figure 6:**
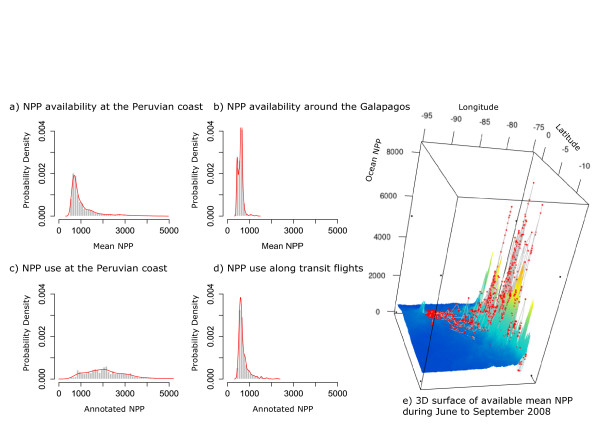
**Probability density histograms and 3D surface plot of Ocean NPP.** Available net primary ocean production (NPP, mg C/m^2^/day) compared to NPP along the tracks of nine Galapagos albatrosses during June to September 2008. Red lines fitted on NPP histograms (left) highlight probability density distributions of NPP use versus NPP availability. Red points connected with gray lines on a 3D surface (right) illustrate the annotated albatross tracks overlaid on the averaged ocean NPP during June to September 2008.

**Figure 7:**
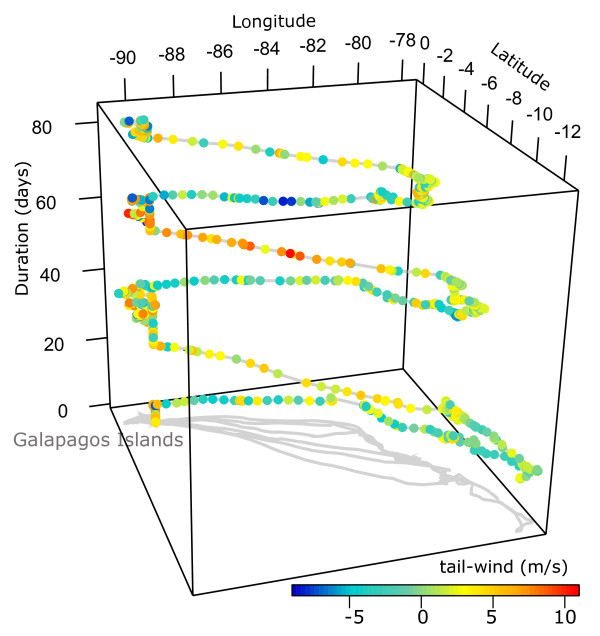
**Space-time-cube illustration of an albatross' flights annotated by tail-wind support.** The track contains 1326 GPS locations of one individual albatross from 23 June to 15 September 2008. The albatross’ outbound flights towards the Peruvian coast are hampered by head winds while the return flights are facilitated by tail-wind assistance.

**Figure 8:**
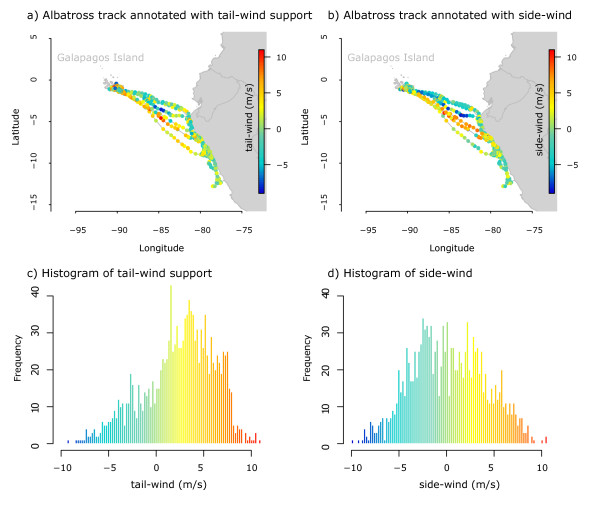
**Map (top) and histogram (bottom) illustration of an albatross’ flights annotated by tail-wind support and side-wind (cross wind).**The track contains 1326 GPS locations of one individual albatross from 23 June to 15 September 2008.

Figures 5, 6, 7, and 8 illustrate the visual exploration tools from the Knowledge Discovery and Visualization service package of *Env*-*DATA*. R code for the generation of these plots is provided in the supporting material (Additional File 1). Figure 5a shows the nine Galapagos Albatross trajectories annotated with 8-day ocean NPP (cf. Function 1, Additional File 1) and Figure 5b shows a gridded geographical area of MODIS chlorophyll-a concentration during one month in the nesting season in 2008, provided as a KML file and plotted using Google Earth. The albatrosses flew to the Peruvian coast to forage where ocean productivity was high. Figure 6 facilitates the comparison of available NPP versus NPP use along flight tracks of the nine albatrosses (the 3D plot is generated using Functions 3, Additional File 1). Figure 6a–b shows the distribution of ocean NPP available at the coastal area of Peru—between 82.5°W, 12°S and 75°W, 4°S—and around the Galapagos Islands—between 95°W, 2.5°S and 90°W, 2.5°N. To illustrate the availability of ocean NPP (Figure 6a–b, Figure 6e), the 8-day NPP datasets are averaged over the period of June–September 2008 in the native grid. Figure 6c–d shows probability density histograms of annotated ocean NPP values along track segments during foraging flight along the Peruvian coast and along flight tracks around the Galapagos, respectively. In addition, Figure 6e provides a 3D visualization of the available NPP versus NPP use along the actual flight tracks (plotted using Function 3, Additional File 1). The NPP histogram constructed from locations along the foraging flight (what was used) illustrates a bias toward high NPP values (Figure 6c), even when compared to the availability near the coast (Figure 6a), which suggests that albatrosses might carefully choose times and locations to forage where NPP is high. However, the similarity between the NPP use and availability histograms around the islands suggests that they do not show preferences for high NPPs along transit flights between the foraging areas and the nesting grounds.

These findings are in accordance with previous studies showing that parents make long-range trips to preferred, productive areas [47].

We use a space-time-cube visualization to illustrate the role of wind on the albatross’ flight patterns (see Figure 7, Function 2, Additional File 1). Recent studies suggest that wind was an important factor in determining migration and short-term flight patterns of pelagic birds [4, 24, 36, 48]. Here, we show how *Env*-*DATA* annotation can assist an investigation of wind dependencies and flow assistance. Figures 7 and 8 show a single albatross trajectory annotated by tail-wind support and side-wind (cross wind), two derived variables (Table 1) computed using wind direction and wind speed and movement direction (flight heading) of the albatross along its flight path, based on the formulation from [24, 36]. The space-time-cube illustrates how wind assistance facilitates the albatross’ flights toward the Galapagos Islands (orange to red colors represent higher wind assistance), while the flights to the coast are often challenged by head wind (aquamarine to blue colors represent wind resistance). The flight pattern in Figures 7 and 8 is characteristic to most other flight tracks in our albatross dataset. As seen in Figure 7 and Figure 8a–b, the albatross repeatedly takes a more northern route to the coast relying mostly on side winds, and then moves south (presumably foraging) before returning to the Galapagos Islands using a tail-wind assisted route (cf. Figure 8a and Figure 8c). The observed clock-wise pattern is in accordance with previous findings [4, 48]. Weimerskirch et. al [48] found that albatrosses prefer tail or side winds and therefore use predictable weather systems to fly in large looping tracks; when going south movements are in a clockwise direction. This enables albatrosses to achieve high speeds while expending little energy. The travel direction towards continental South America and back to the Galapagos undertaken by waved albatross means they almost always have side-winds (cf. Figure 8b and Figure 8d).

## Conclusions

We presented the Environmental-Data Automated Track Annotation (*Env*-*DATA*) System, an openly available portal within Movebank (http://www.movebank.org), and illustrated how the system assists the discovery of environmental conditions associated with animal movements. As compared to the existing RNCEP package, that provides access to, organization, and visualization of atmospheric NCEP/NCAR datasets, the *Env*-*DATA* annotation service streamlines the co-registration of animal tracking data with a diverse range of environmental variables obtained from satellite remote sensing products and global reanalysis models including the MODIS ecological, ocean, land cover and land use data sets, the NCEP/NCAR and ECMWF weather reanalysis datasets, high-resolution Digital Elevation Models (DEMs), and ecological and human-socioeconomic reanalyses (e.g. the Population Density Grid). This project has overcome the numerous technical and methodological challenges in order to enable processing of a large array of remote sensing, weather and geographical data for the analysis of animal movement tracks:

-  optimizing storage and retrieval times for a very large dataset of environmental variables from multiple data providers,
-  applying effective interpolation techniques in order to maintain the link between animal tracks and their embedding environment in space and time,
-  applying suitable spatiotemporal indexing strategies for data retrieval, and
-  maintaining a large database of remote sensing data.

In addition, our system is intended as a general tool that can be used by researchers at all levels of technical ability for a wide range of animal movement data types and research questions. Thus additional challenges were:

-  establishing linkages between heterogeneous environmental and movement data, obtained from various sources, collected in different spatial and temporal resolutions and scales; and
-  developing a user-friendly interface within Movebank to allow users to browse, access documentation about variables and source datasets, and select and request annotated data.

In addition to the main annotation service, we are currently developing the Knowledge Discovery and Visualization Service and the Track Simulation Service within the *Env*-*DATA* System. In future releases, we aim to exploit deterministic and probabilistic computational GIS methods, spatiotemporal data mining techniques, and well-known statistical approaches, with the underlying goal to discover patterns and structures among the movements of animals.

### Availability of supporting data

The albatross dataset supporting the results of this article is accessible through DOI: 10.5441/001/1.3hp3s250 and can also be viewed at http://www.movebank.org in the study “Galapagos Albatrosses”. The R scripts used for generating Figures 5–8 in this article are provided in Additional File 1.

## Electronic supplementary material

Additional file 1: **R scripts of visualization tools.** (DOCX 91 KB)
