# Supplementary material for: The environmental-data automated track annotation (Env-DATA) system: linking animal tracks with environmental data
Source: Mov Ecol. 2013 Jul 3;1(1):3. doi: 10.1186/2051-3933-1-3 (PMC4337772; doi:10.1186/2051-3933-1-3)
Supplement: Supplementary file 1 — Additional file 1: R scripts of visualization tools. (DOCX 91 KB) [file 40462_2012_3_MOESM1_ESM.docx]

# Additional Files

## Additional File 1 - R scripts of visualization tools

The following R functions were used to illustrate annotated movement tracks and the section Case Study: Galapagos Albatross (*Phoebastria irrorata*). The variables used in the functions are defined as follows:

- “move”: represents a set of animal locations as a SpatialPointDataFrame (“move” contains coordinates—longitude and latitude—timestamps, and other attributes such as annotated variables obtained from *Env-DATA*)
- “variable”: environmental variable name existing as a column in *move.* (e.g. oceanNPP)
- “lonlim”: a vector of min and max longitudes (e.g. lonlim = c(-95,-75))
- “latlim”: a vector of min and max latitudes (e.g. latlim = c(-14,10) )
- “envdata”: is a data.frame containing three columns in *long* format: x (e.g. longitude) , y (e.g. latitude), and z (e.g. value)

### Function 1 - 2D plot of a movement track colored based on values of a variable

function(move, variable, latlim, lonlim)

{

require(maptools)

require(sp)

require(field)

vCol<- which(colnames(move@data)==variable)

temp<- levels(as.factor(as.character(unique(move@data[,vCol]))))

temp<-as.character(temp[!is.na(temp)])

zr=range (as.numeric(temp))

# generate a color scale

colorpalette <- colorRampPalette(c("blue3","cyan3","aquamarine3","yellow","orange","red"))

mycol=colorpalette(length(temp))

# plot underlying map

data(wrld_simpl)

plot(wrld_simpl, ylim=latlim, xlim=lonlim, col="lightgray", border="gray", axes=F)

# plot the track as a gray ployline

points(coordinates(move), type="l", col="gray")

# plot track points colored using a color scale based on the variable values (high values in Red, low values in Blue)

for(i in 1:length(temp))

{

plot (move[move@data[,vCol]==temp[i],],

pch=20,add=TRUE, col=mycol[i], border="gray", axes=F)

}

# plot color scale legend

image.plot( legend.only=TRUE, zlim= zr, col=mycol, horizontal=F, legend.width = 1, legend.mar = NULL, legend.shrink=0.8, cex.axis=0.7)

}

### Function 2 - Space-time-cube plot of a movement track colored based on values of a variable

function(move, variable, lonlim, latlim)

{

require(maptools)

require(sp)

require(field)

require(rgl)

move$dtime<- difftime(move$timestamp2,trajectory[1,]$timestamp2, units='days')

vCol<- which(colnames(move@data)==variable)

temp <- as.numeric(levels(as.factor(move@data[,vCol])))

temp<-as.character(temp[!is.na(temp)])

# generate a color scale

colorpalette <- colorRampPalette(c("blue3","cyan3","aquamarine3","yellow","orange","red"))

mycol=colorpalette(length(temp))

open3d()

# plot 3D track as a gray polyline ploylineplot3d(coordinates(move)[,1],coordinates(move)[,2], dtime, col="lightgray", type="l")

# plot underlying 2D track as a gray polyline

plot3d(coordinates(move)[,1],coordinates(move)[,2], 0, ticktype="simple", col="lightgray", type="l", add=T, axes=F)

# plot track points colored using a color scale based on the variable values (high values in Red, low values in Blue)

for(i in 1:length(temp))

{

plot3d(coordinates(move[move@data[,vCol]==temp[i],])[,1],coordinates(move[move@data[,vCol]==temp[i],])[,2], move[move@data[,vCol]==temp[i],]$dtime, type="p", size=5, col=mycol[i], add=TRUE, axes=F)

}

}

### Function 3 - 3D surface plot of a variable overlaid by movement tracks

function(envdata, move,variable){

# this function is written using “plot_rgl_model_a” function from http://stackoverflow.com/a/12779434

require(reshape2)

require(rgl)

envdata <- envdata[order(envdata[, 1], envdata[, 2]), ]

orig_names <- colnames(envdata)

colnames(envdata) <- c("x", "y", "z")

xlim <- zr=range (as.numeric(envdata$longitude))

ylim <- zr=range (as.numeric(envdata$latitude))

zlim <- c(-1, max(envdata$variable, na.rm = T)) # -1 is used instead of missing values (e.g. -9999)

l <- list (x = xlim, y = ylim, z = zlim)

xyz <- do.call(expand.grid, l)

x_boundaries <- xyz$x

y_boundaries <- xyz$y

z_boundaries <- xyz$z

# reshape envdata into a wide format to be used in rgl.surface()

envdata[, 2] <- as.character(envdata[, 2])

envdata[, 3] <- as.character(envdata[, 3])

wide_form <- dcast(envdata, y ~ x, value.var = "z")

wide_form_values <- as.matrix(wide_form[, 2:ncol(wide_form)])

x_values <- as.numeric(colnames(wide_form[2:ncol(wide_form)]))

y_values <- as.numeric(wide_form[, 1])

wide_form_values <- wide_form_values[order(y_values), order(x_values)]

wide_form_values <- as.numeric(wide_form_values)

x_values <- x_values[order(x_values)]

y_values <- y_values[order(y_values)]

ind<- which(wide_form_values < 0)

wide_form_values [ind]<- -1

zlim <- range(wide_form_values)

zlen <- zlim[2] - zlim[1] + 1

# generate a color scale

colorpalette <- colorRampPalette(c("blue3","cyan3","aquamarine3","yellow","orange","red"))

colorlut <- c("white", colorpalette(6000)) # white is used for missing data

col <- colorlut[ wide_form_values-zlim[1]+1 ] # assign colors to variable values for each point

open3d()

plot3d(x_boundaries, y_boundaries, z_boundaries,

box = T, col = "black",xlab = "", ylab="", zlab="")

rgl.surface(z = x_values, x = y_values, y = wide_form_values, coords = c(2,3,1), color = col, alpha = 1.0, lit = F)

# plat track points in red connected with gray polylines

plot3d(coordinates(move)[,1],coordinates(move)[,2],move$variable, type="l", col = "gray", add=TRUE)

points3d(coordinates(move)[,1],coordinates(move)[,2], move$variable, col = "red")

}
